# Predicting Treatment Outcomes in Patients with Drug-Resistant Tuberculosis and Human Immunodeficiency Virus Coinfection, Using Supervised Machine Learning Algorithm

**DOI:** 10.3390/pathogens13110923

**Published:** 2024-10-24

**Authors:** Mojisola Clara Hosu, Lindiwe Modest Faye, Teke Apalata

**Affiliations:** 1 Department of Laboratory Medicine and Pathology, Faculty of Medicine and Health Sciences, Walter Sisulu University, Private Bag X5117, Mthatha 5099, South Africa; fayelindiwe@yahoo.com (L.M.F.); ruffinapalata@gmail.com (T.A.); 2 National Health Laboratory Service (NHLS), Mthatha 5100, South Africa

**Keywords:** DR-TB, DR-TB/HIV coinfection, treatment outcomes, machine learning, supervised learning algorithm

## Abstract

Drug-resistant tuberculosis (DR-TB) and HIV coinfection present a conundrum to public health globally and the achievement of the global END TB strategy in 2035. A descriptive, retrospective review of medical records of patients, who were diagnosed with DR-TB and received treatment, was conducted. Student’s *t*-test was performed to assess differences between two means and ANOVA between groups. The Chi-square test with or without trend or Fischer’s exact test was used to test the degree of association of categorical variables. Logistic regression was used to determine predictors of DR-TB treatment outcomes. A decision tree classifier, which is a supervised machine learning algorithm, was also used. Python version 3.8. and R version 4.1.1 software were used for data analysis. A *p*-value of 0.05 with a 95% confidence interval (CI) was used to determine statistical significance. A total of 456 DR-TB patients were included in the study, with more male patients (n = 256, 56.1%) than female patients (n = 200, 43.9%). The overall treatment success rate was 61.4%. There was a significant decrease in the % of patients cured during the COVID-19 pandemic compared to the pre-pandemic period. Our findings showed that machine learning can be used to predict TB patients’ treatment outcomes.

## 1. Introduction

The global attempts to control tuberculosis (TB) notwithstanding, TB is positioned as the top contributor to mortality from a single infectious agent and is a grave public health dilemma. The WHO reported that an estimated 10.6 million people contracted TB in 2022, with 1.3 million patient fatalities [1,2,3]. Tuberculosis (TB) is an infectious disease caused by *Mycobacterium tuberculosis* [4,5]. Drug-resistant TB (DR-TB) occurs when these bacteria become resistant to the drugs used to treat TB, which can no longer destroy the TB germ [5]. Among cases of DR-TB in 2019, people who were categorized as TB monoresistant, which is resistance displayed to isoniazid, termed INH-R, were over a million in number, while patients who demonstrated resistance to rifampicin, RR, were about half a million, with multidrug-resistant TB (MDR-TB) accounting for 78% of this estimate [2,6,7]. DR-TB is associated with diagnostic and therapy intricacies, longer therapy regimens, morbidity, and fatality, making it challenging to deal with. The WHO report in 2022 indicated that only about 40% of patients with DR-TB accessed treatment [3]. MDR-TB happens when resistance to two main antituberculosis medications, isoniazid and rifampicin, is displayed and associated with prolonged hospitalization and increased mortality [1,8,9]. Globally, RR-TB and MDR-TB represent 3.3% and 18% of incident cases and previously treated cases, respectively; hence, the emergence of MDR-TB seriously threatens the END TB strategy of the WHO to reduce TB deaths by 2030 [7].

TB control has recorded good progress in recent times. However, the emergence of MDR-TB in many regions has made the gains almost inconsequential, with a global treatment success rate (TSR) pegged at 59% [10]. This public health crisis is aggravated by coinfections, comorbidities, and an increase in the pool of latent infection [11]. In 2021, global deaths from TB recorded 1.6 million people, out of which 11% were HIV coinfections. In the USA, 7882 cases were reported, and an estimated 13 million people live with latent tuberculosis infections [4]. The Asia–Pacific region, which contributes more than half of all tuberculosis (TB) cases worldwide, reported a TB/HIV coinfection rate of 6.3% in 2013 [12]. In the African region, data on TB/HIV coinfection showed varied values ranging between 2.9% and 72.3%, with a pooled prevalence of 23.5% [13]. The national average rate for Ghana is 22% [14], while a study in Ethiopia reported 25% [15]. However, a hospital-based cross-sectional study conducted from May 2022 to February 2023 in Northwest Amhara, Ethiopia, reported an overall proportion of TB with an HIV coinfected rate of 19.8% [16]. This figure is, however, higher than the global estimate of 15% and much lower than the African regional estimate of 36% [17]. Sub-Saharan Africa (SSA) harbors the highest burden of coinfection, with 71% of global cases [18], while in South Africa, approximately 180,000 incident TB cases are people with HIV coinfection [19]. In 2015, the average TB/HIV coinfection rate across South Africa was 56.7%, with Gauteng, Mpumalanga, and KwaZulu-Natal (KZN) reporting the highest coinfection rates at 68.4%, 68.1%, and 63.6%, respectively. Eastern Cape, Northern Cape (NC), and Western Cape had far lower rates of HIV coinfection at 45.2%, 41.1%, and 38.5%, respectively. Across the districts, TB/HIV coinfection rates varied from a high of 71.2% in uThukela (KZN) to a low of 25.0% in Namakwa (NC), with Khayelitsha in the Western Cape at a rate of 50.5% [20,21].

Despite the scale-up of antiretroviral therapy (ART), TB remains the main cause of HIV-related morbidity and mortality worldwide [3]. The correlation between TB and HIV infection fast-tracks the advancement of disease in people living with HIV(PLHIV), thereby contributing to the failure of TB-control programs to reach successful treatment targets, particularly in high-burden countries. Furthermore, TB and HIV coinfection facilitates the acquisition of MDR and the extensively drug-resistant TB (XDR-TB) strains. There is a higher risk (19-fold) of developing active TB in PLHIV, especially with a CD4 count lower than 200/cm^3^, compared with people who are HIV-negative [18]. Despite the introduction of new ART and anti-TB drugs, the dual management of TB and HIV coinfection represents an intricate clinical conundrum that requires a logical approach to curtail treatment failure and minimize morbidity and fatality [22]. Safety concerns of co-administration in therapy include drug–drug interactions that result in subtherapeutic concentrations of ART and anti-TB drugs; this results in impaired efficacy, development of overlapping toxicities, and immune reconstitution inflammatory syndrome (IRIS) [22].

Several targets [23] to limit the co-epidemics of TB and HIV and the emergence of MDR-TB were set in the 2011–2015 Global Plan to End TB [24]. These targets include 100% of TB patients knowing their HIV status, 100% of HIV/TB coinfected patients receiving antiretroviral therapy, and newly enrolled patients in HIV care programs with latent TB infection receiving isoniazid as a form of preventive TB therapy [23]. The target to detect and treat all MDR-TB cases with second-line TB medications was set in 2015, with a 75% treatment success rate. None of these targets have been attained in SSA [25]. The 2030 End TB strategy now seeks to end the global TB epidemic, with targets of a TB mortality reduced by 95% and TB incidence reduced by 90% compared to 2015 [23].

A computerized predictive model is a statistical technique using machine learning (ML) and data mining to predict and forecast possible future outcomes with the help of an existing dataset. Liu et al. [26] defined ML methods as the method of generating predictive models based on patterns of learning features from data, which predict new data or outcomes through the constructed model. These methods are subdivided into three types: supervised learning, unsupervised learning, and reinforcement learning [27,28]. Supervised learning uses categorized data as a training dataset in its algorithm, where the outcome of interest is defined [27]. The decision tree algorithm uses supervised learning to predict and classify data [29]. The trees are diagram-like, resembling flowcharts, with a root node for data inquiry [30]. ML has been found useful in the medical field for clinicians in practice and occasionally performs better than human expertise [31]. In the clinical space, ML has also been used to analyze biological datasets for cancer and HIV/AIDS research, including in drug discoveries [32]. ML systems have been developed in several fields of medicine, including radiology, for interpreting chest X-ray scans or magnetic resonance imaging for diagnostic purposes [33,34]. A first-of-its-kind independent approval was issued by the FDA in 2018 to IDx, an ML system, for detecting diabetic retinopathy [35]. In infectious diseases, research, drug development, or clinical microbiology remains the focus of most ML systems. These include HIV genotyping and the prediction of susceptibility to antiretroviral (ARV) drugs [36], the analysis of bacterial genomes and the improvement of resistance prediction [37,38], the discovery of vaccines and antibacterial drugs [39,40], and epidemic patterns for surveillance purposes [41,42]. Évora et al. [43] used ML methods for the identification of MDR-TB patients in the Brazilian city of Rio de Janeiro. The most common predictive models include decision trees, regressions (linear and logistic), and neural networks. ML techniques such as artificial neural networks, random forests, and support vector machines have employed clinical and genotypic data to provide useful predictions about patient outcomes [31,44]. Such methods have proven viable in selecting new regimens [31,45]. Moreover, including clinical data in training a prediction model enhances its accuracy [31]. However, limited research is available on the efficacy of ML techniques for predicting treatment outcomes in our setting.

Given these interrelated disease conditions, analyzing treatment efficacy for patients burdened with DR-TB and HIV requires an integrated approach. It involves evaluating how each condition affects the others and determining the most effective strategies for integrated care. This study aims to analyze treatment efficacy (treatment outcomes and treatment success rate) in patients burdened with DR-TB/HIV coinfection and to analyze factors associated with successful treatment outcomes among patients in the OR Tambo. Moreover, this study uses a machine-learning algorithm to predict and determine the risk factors associated with DR-TB disease.

## 2. Methods

### 2.1. Study Design, Setting, and Population

A descriptive, retrospective review of medical records of DR-TB enrolled in healthcare facilities in OR Tambo between January 2018 and December 2020. The Eastern Cape Province (ECP) is a predominantly rural province and the third biggest province out of nine provinces in South Africa, with a population of approximately 7 million [46]. O. R. Tambo district is one of the 7 districts of the ECP located on the coastline, with a population of approximately 1.4 million. It is made up of five (5) local municipalities, with the seat of administration in Mthatha. The predominant language spoken is isiXhosa. The study participants were drawn from selected five healthcare facilities (HCF) in OR Tambo district municipality in the ECP of South Africa. These healthcare facilities were coded as HCF 1–5.

### 2.2. Data Collection

Data from the medical records of DR-TB patients who began treatment between 2018 and 2021 were recorded. All patients diagnosed with any type of DR-TB during the study period were included. The data collected included sociodemographics, clinical data, and treatment outcomes. Anti-TB drug sensitivity test results were categorized into different classes of drug resistance. According to the 2018 South Africa TB Treatment Guidelines [47], all TB patients were screened for HIV, and patients coinfected with TB and HIV were started on ART as soon as possible, usually within the first two weeks of TB treatment. Cotrimoxazole preventive therapy (CPT) was provided to prevent opportunistic infections in coinfected individuals. Patients with MDR-TB were given injectable agents such as kanamycin or amikacin alongside fluoroquinolones (levofloxacin or moxifloxacin) and other second-line drugs. Shorter treatment regimens of 9–12 months were introduced in certain cases of MDR-TB based on patient response and resistance profiles. For patients with pre-XDR-TB and XDR-TB, more intensive treatment, often including newer drugs like bedaquiline and delamanid, was used in cases of resistance to second-line drugs. The ART regimen used for HIV-positive patients is a fixed-dose combination (FDC) comprising tenofovir (TDF) 300 mg + lamivudine (3TC) 300 mg + DTG 50 mg (TLD). TLD is prescribed for clients ≥ 35 kg and ≥10 years of age [48].

The CD4 cell count is used to assess the progression of HIV disease, including the risk of developing opportunistic infection, and guides preventive treatment. The normal range of CD4 count is from 500 to 1500 cells/mm^3^ of blood, and it progressively decreases over time in persons who are not receiving or not responding well to ART. If the person’s CD4 cell count falls below 200, their immunity is severely compromised, leaving them susceptible to infections and death. A CD4 count range between 200 and 499 is categorized as moderately immunocompromised, while someone with a CD4 count below 200 is described as having an advanced HIV disease (AHD) [49]. The viremic titer was classified into three using the following cut-offs: Virological Failure (Viral load ≥ 1000), low-level viremia (viral load 50–999), and suppressed (viral load < 50). The BMI thresholds proposed by the WHO were used for categorization, namely underweight (<18.5 BMI), normal weight (18.5–24.9 BMI), overweight (25–29.9 BMI), and obese (≥30 BMI) [50]. The presence of acute and chronic comorbidities was recorded.

### 2.3. Statistical Analysis

Data on the study participants were described using absolute frequencies and percentages for categorical variables; measures of central tendency (mean, median, range) and dispersion (standard deviation and interquartile range) were used for continuous variables. Student’s *t*-test was performed to assess differences between two means and ANOVA between groups. Either the Chi-square test with and without trend or Fischer’s exact test was used to test the degree of association of categorical variables. ANOVA assumes that the data follow a normal distribution and has homogeneity of variance. If the initial tests (Chi-Square or ANOVA) show significant results, post-hoc pairwise comparison tests were used to identify which specific groups are significantly different from each other. Logistic regression was used to determine predictors of DR-TB treatment outcomes. A decision tree classifier, which is a supervised machine learning algorithm, was also used. Decision trees were used to split data based on the value of input features (e.g., age, income, comorbidities) to make predictions (e.g., whether treatment will be successful or unsuccessful).

Several steps in using a decision tree include preparing the data, training the model, and evaluating its performance. Suppose we have data on 100 patients with each patient’s record, including their age, gender, residence, education, annual income, DR-TB, and comorbidities. We first cleaned the dataset, ensuring no missing values, and the feature predicting the treatment outcome is split into training and test sets as 70/30 or 80/20 split. We chose the model type, which is a simple decision tree classifier. Parameters like the maximum depth of the tree and the minimum number of samples required to split a node were set up, and the decision tree classifier was trained with the training data with the training process involving finding patterns in the data that lead to decisions at each node in the tree. Using the test set, we evaluated the model’s performance with metrics such as accuracy, precision, recall, and F1-score. The decision tree was visualized to understand how decisions were made.

All patients diagnosed with DR-TB during the study period were included. One of the strongest ways to carry out prediction is using machine learning. The choice of variables selected for inclusion in our machine learning model was conducted in three ways. Firstly, we conducted a comparative assessment between the baseline characteristics of DR-TB patients in the OR Tambo district (n = 456 enrolled) and the underlying general population characteristics within the same district (n = 1,514,306 according to census data). The results suggest that DR-TB disproportionately affects individuals aged 20–59 years compared to the general population, while those under 20 and over 60 are less affected (*p* < 0.0001). The analysis shows a significant difference in the distribution of males and females between the DR-TB patients and the general population. Males were significantly overrepresented in the DR-TB population, while females were underrepresented compared to the general population (*p* = 0.00003). Secondly, variables obtained from the logistic regression model predicting treatment outcomes were selected. These included HIV, one of the comorbidities, gender, and education level. Thirdly, evidence from the literature also supported our choice of these variables [7,51,52,53,54].

The decision tree splits the dataset based on threshold values for the features at each node. Gini impurity, which is a measure of how “pure” a node is or how mixed the classes (successful vs. unsuccessful) are at that node, was determined. For each split, the algorithm tries to minimize the Gini impurity, meaning it looks for the feature and threshold that creates the most homogeneous nodes (e.g., where most patients either succeed or fail). Splits were made based on variables selected to maximize the difference between the two classes (successful vs. unsuccessful treatment) after the split. The algorithm recursively partitions the dataset, starting at the root (top) node and continuing until a stopping criterion is met (e.g., maximum tree depth or minimum node size). At each step, the decision tree algorithm selects the feature and value that produces the largest information gain, meaning it reduces uncertainty (measured by Gini impurity) the most. Potential biases such as overfitting and selection bias, variance, bias toward certain splits, and imbalanced data occurring in the dataset were planned for during the modeling process to significantly improve the performance and reliability of our decision tree model. In decision trees, pruning techniques were applied to avoid overfitting by removing branches that add little predictive power or fit too closely to the training data. This improves the model’s ability to generalize new, unseen data. Ensemble Techniques were also used to reduce variance and improve robustness by averaging multiple decision trees. Adjusting class weights was performed to handle imbalanced data more effectively, and cross-validation was employed to ensure that the model’s performance was stable across different subsets of the data.

Python version 3.8. and R version 4.1.1 software were used. A *p* < 0.05 was considered to be significant.

## 3. Results

### 3.1. Sociodemographic and Clinical Characteristics of Study Participants

A total of 456 participants with records of DR-TB patients were assessed. The study had more male patients (n = 256, 56.1%) than female patients (n = 200, 43.9%). The study participants’ age distribution ranged from 1 to 86 years, with a mean age of 37.5 (SD ± 14.9) years. The median age (50th percentile) and 75th percentile were 36 and 47 years, respectively. The proportion of patients aged 19–35 was 41.6%, followed by those aged 36–50 with 31.9%. HIV/TB coinfection was observed in 281 (61.6%) patients. Those above the age of 66 comprised 4.7%, whereas 51–65 constituted 14.6%. Table 1 presents the sociodemographic and clinical characteristics of the patients.

Table 2 shows the type of DR-TB patients from 2018 to 2020, categorized by age. Most of the DR-TB cases were categorized as RR-TB with 45.0% prevalence, while the proportion of MDR-TB was 42.5%. pre-XDR and XDR-TB only accounted for 8.7%. The 19–35 age group had the highest concentration of RR-TB cases at 41.5%, followed by the age group 36–50 with 32.7% prevalence. A similar pattern was observed with the MDR-TB cases, with the 19–35 age group accounting for 43.3%, followed by the age group 36–50 with 32% prevalence.

**Figure 1:**
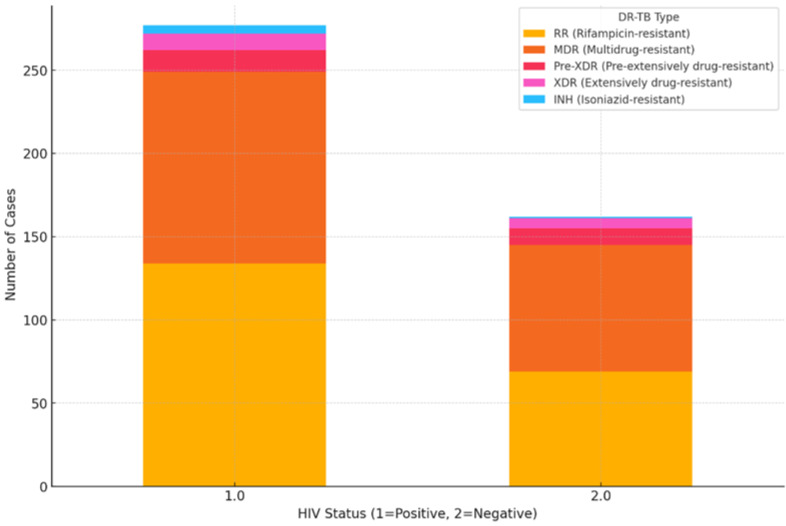
Distribution of DR-TB stratified by HIV status.

Figure 1 is a graphical illustration of the categorization of DR-TB by HIV status. The results of the Chi-square tests for each DR-TB type reveal that for each DR-TB type, the differences in distribution between HIV-positive and HIV-negative patients are not statistically significant. This suggests that while there are more cases of certain DR-TB types in HIV-positive patients, the differences observed in the dataset may be due to random variation rather than a systematic association between HIV status and DR-TB type.

#### 3.1.1. Categorization of the Patients According to Their CD4 Lymphocyte Count and Viral Load

Sixty-seven (67) patients were in the severely immunocompromised category, while the moderately immunocompromised patients were thirty-three (33). Those with a normal CD4 count comprised sixteen (16) patients. Three viral load categories, including virological failure with 78 patients, low-level Viraemia with 32 patients, and suppressed with six patients.

**Figure 2:**
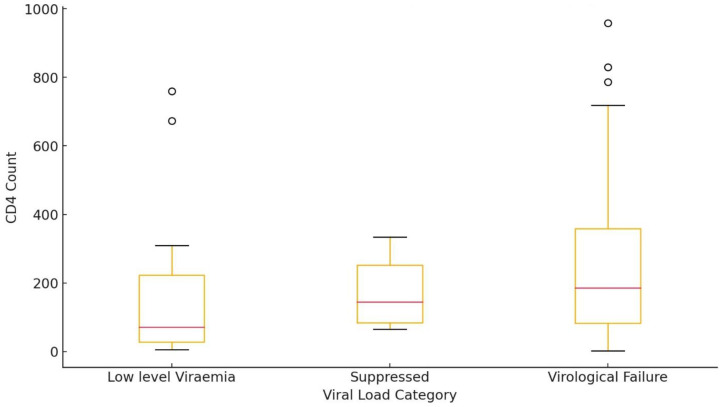
CD4 count distribution stratified by viral load.

The box plot for CD4 count distributions across the viral load categories is indicated in Figure 2. Patients categorized in the virological failure group have the broadest range of CD4 counts, from a minimum of 2 to a maximum near 958. This group’s median (50th percentile) CD4 count is 186, indicating that half of the patients have a CD4 count below this value. The interquartile range (IQR), between the 25th and 75th percentiles, is broad, showing high variability in CD4 counts among patients. This variability might indicate that immune response varies significantly among those with virological failure.

Patients categorized in the low-level viremia have CD4 counts more concentrated, ranging from 6 to 759, lower than the virological failure group, meaning the immune systems of many patients in this group may be more compromised than those in virological failure.

The suppressed category has the smallest range from 65 to 334 CD4 lymphocyte counts, with a relatively high median CD4 count of 145, suggesting that patients with suppressed viral loads generally have stronger immune systems when compared to the other groups. The smaller range and IQR indicate that patients in this group are more homogenous regarding their immune response, reflecting a more stable or controlled condition.

Patients categorized in the virological failure group show the most variation in CD4 counts, with some maintaining relatively high counts despite their viral load. The low-level Viremia patients have lower CD4 counts, reflecting more compromised immune function, while the Suppressed patients have higher and more consistent CD4 counts, indicating better immune recovery or control.

#### 3.1.2. Body Mass Index (BMI) Categorization

The BMI values range from a minimum of 11.0 to a maximum of 47.0, with a mean BMI of 22.08 (±5.47) standard deviation.

**Figure 3:**
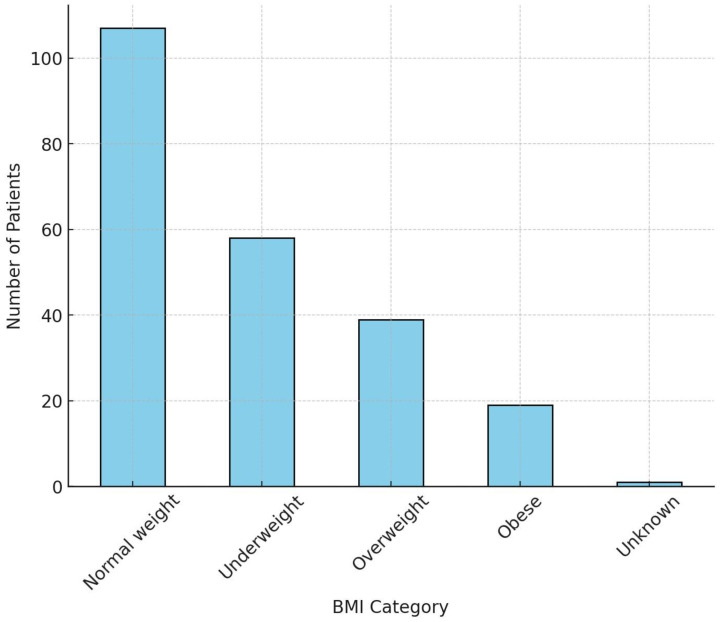
Distribution of BMI categories in the study’s population.

The bar chart (Figure 3) shows the distribution of patients across different BMI categories.

Normal weight represents the largest category, with 107 patients. It indicates that most patients fall within the normal weight range, suggesting their BMI is within the standard healthy range. Fifty-eight (58) patients were categorized as underweight, a significant portion of the population. This could be a concern in the context of HIV-positive patients, as underweight individuals may have a higher risk of complications due to poor nutritional status and lower immune response. Thirty-nine (39) patients fall into the overweight category. While this group is smaller, it is important to monitor it, as being overweight could increase risks for other health issues like heart disease or diabetes. Only 19 patients were categorized as obese. Although this group is the smallest, obesity is typically associated with higher health risks, including metabolic disorders, which could complicate treatment or disease progression.

#### 3.1.3. Presence of Chronic Comorbidities

Common chronic conditions in our study population included hypertension (HTN), high blood pressure, Type 2 Diabetes (T2DM), a chronic condition affecting blood sugar regulation, epilepsy, a neurological condition marked by recurrent seizures, mental illness, chronic mental health disorders such as depression, anxiety, and bipolar disorder, liver disease, chronic liver conditions such as cirrhosis or hepatitis, and hearing loss, a chronic hearing impairment or loss of hearing ability.

### 3.2. Treatment Outcomes of DR-TB

**Figure 4:**
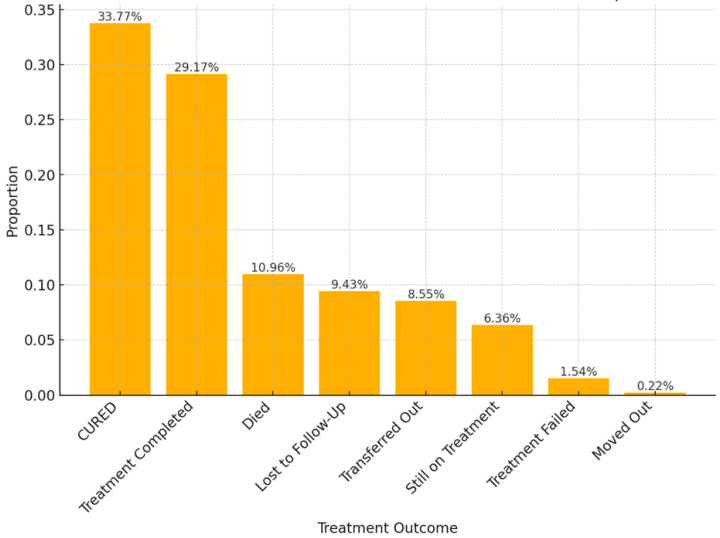
Treatment outcomes of patients with DR-TB at selected hospitals in the OR Tambo district municipality between January 2018 and December 2020.

The final DR-TB treatment outcome results recorded for the patients cured (n = 153, 33.77%), treatment completed (n = 126, 29.17%), and those identified as lost to follow-up (n = 43, 9.43%); those who died (n = 50, 11%), those who moved out (n = 1, 0.2%), transferred out (n = 37, 8.55%) and those who experienced treatment failure (n = 7, 1.54%) and those who were still on treatment (n = 29, 6.36%) (Figure 4).

### 3.3. Treatment Outcomes of DR-TB Comparing COVID-19 Pre-Pandemic (2018–2019) and Pandemic (2020–2021) Periods

There was a significant decrease in patients cured during the COVID-19 pandemic (42 cases) compared to the pre-pandemic period (111 cases). Similarly, the number of completed treatments dropped from 95 cases pre-pandemic to 31 during the pandemic. The number of patients lost to follow-up (LTFU) also decreased significantly during the pandemic, possibly due to fewer patients starting treatment or challenges in tracking patients. The number of deaths remained relatively stable, slightly decreasing during the pandemic.

**Figure 5:**
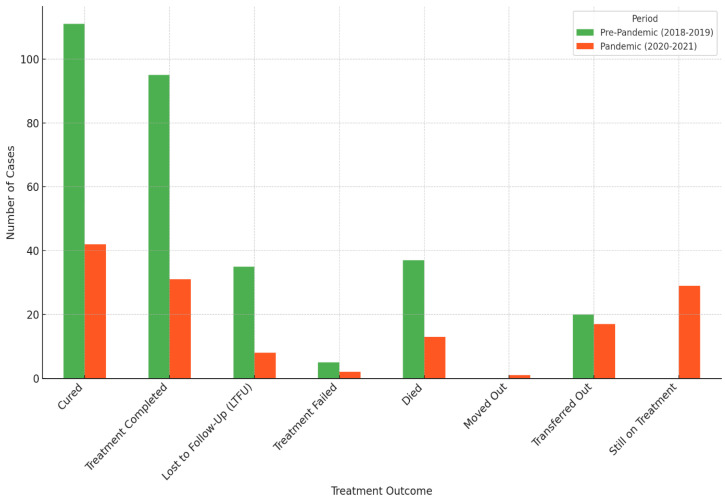
Pre-pandemic versus pandemic treatment outcomes in the study’s population.

Figure 5 below presents a graphical illustration comparing treatment outcomes between the pre-pandemic and pandemic periods. It highlighted the significant differences in the number of cases for each outcome, with the pandemic period showing a notable decrease in cured cases and completed treatments.

### 3.4. The Trend of Treatment Outcomes over Time

Cured and Treatment Completed: The highest number of cases where treatment was completed or patients were cured occurred in 2019. However, there was a noticeable drop in 2020 and almost no cases in 2021, which correlates with the overall decrease in treatment starts.

Lost to Follow-Up (LTFU): The number of patients lost to follow-up remained relatively stable in 2018 and 2019, with a slight drop in 2020 and no cases in 2021.

Died: The number of deaths remained consistent across the years, peaking in 2020. This could suggest worsening cases or other external factors impacting patient outcomes.

Transferred Out: There was a decrease in patients transferred out in 2019, followed by a slight increase in 2020 and 2021.

**Figure 6:**
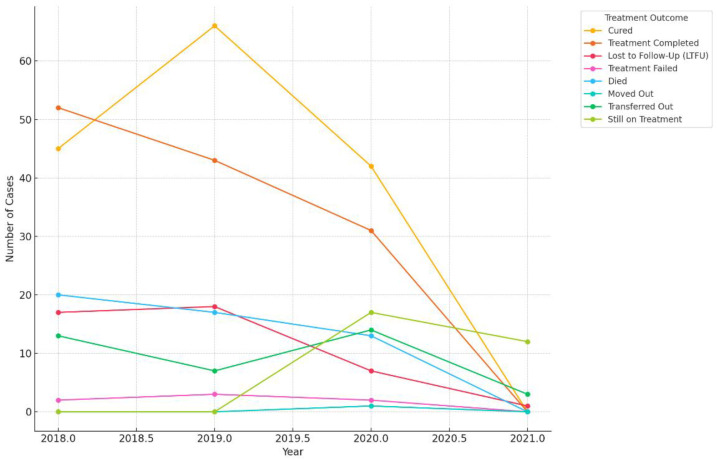
Treatment outcomes trend in the study’s DR-TB population.

Still on Treatment: Patients still on treatment increased significantly in 2020 and 2021, indicating that treatments might have been prolonged or delays were experienced in completing treatments (Figure 6).

### 3.5. Association Between DR-TB and Treatment Outcomes

**Figure 7:**
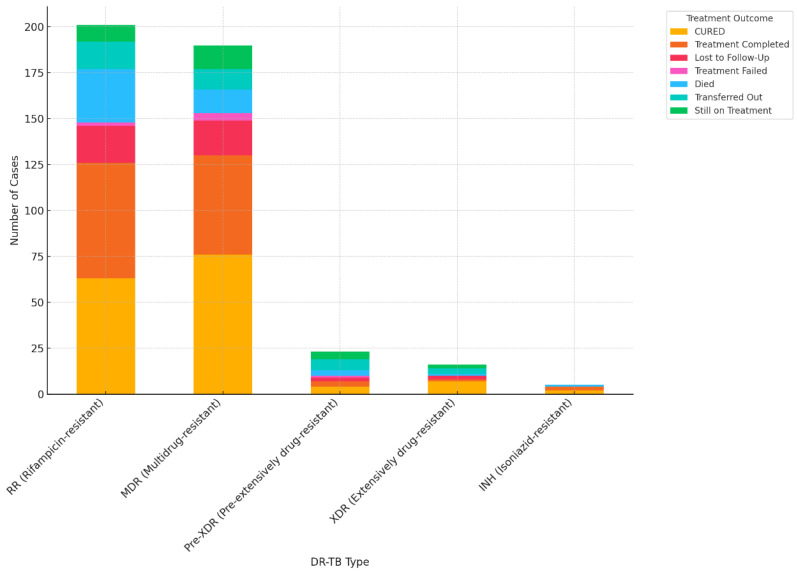
Treatment outcomes according to DR-TB type.

The association between DR-TB type and treatment outcomes is statistically significant (*p*-value = 0.028) (Figure 7). The results indicate a significant association between the type of DR-TB and the treatment outcome. Different types of drug resistance in TB are associated with different treatment outcomes, and these variations are statistically significant.

**Figure 8:**
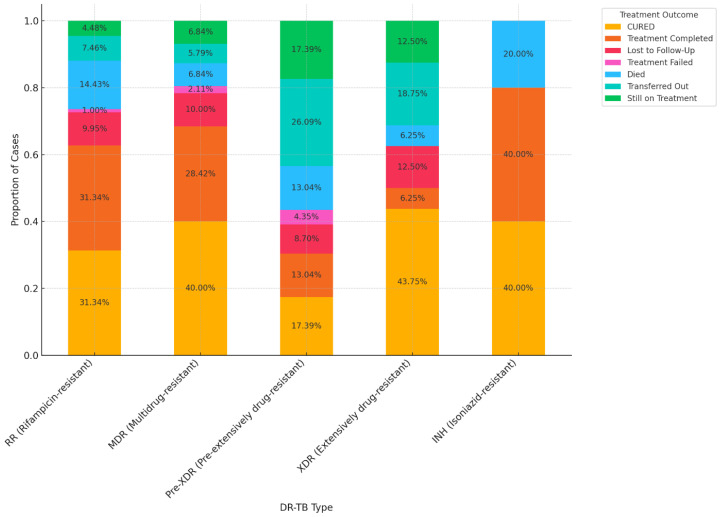
Proportion of treatment outcomes stratified by DR-TB.

The proportion of cases for each treatment outcome within each DR-TB type is displayed in Figure 8. The percentages within each bar segment represent the relative frequency of each outcome, providing a clear comparison of how treatment outcomes differ by DR-TB type.

**Figure 9:**
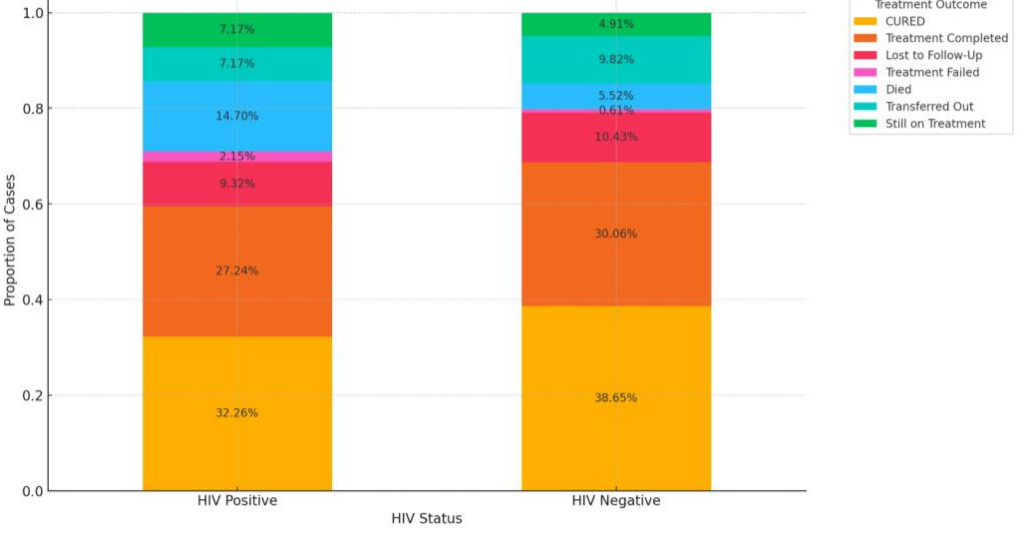
Proportion of treatment outcomes stratified by HIV status.

Figure 9 shows the proportion of treatment outcomes by HIV status. The association using the chi-square test indicates a *p*-value of 0.051. The *p*-value is slightly above the common significance threshold of 0.05, suggesting that the association between HIV status and treatment outcome is marginally not statistically significant, though very close to the threshold. In other words, there may be some association, but the evidence is not strong enough to confidently reject the null hypothesis. While there appears to be some relationship between HIV status and treatment outcomes, the evidence is not strong enough to conclude a statistically significant association at the 0.05 level. However, given that the *p*-value is close to 0.05, this result could still be considered noteworthy, and further investigation with a larger sample size might be warranted.

From the above Figure 9, a higher mortality was recorded with HIV-positive patients. The higher proportion of deaths among HIV-positive patients highlights the increased vulnerability of this group during TB treatment. The higher rate of “Lost to Follow-Up” among HIV-positive patients suggests potential difficulties in maintaining treatment adherence or access.

Overall Treatment Success: Although the proportions of Cured and Treatment Completed are substantial in both groups, HIV-negative patients generally fared slightly better, with higher success rates and lower mortality.

### 3.6. Impact of HIV Coinfection on Treatment Outcomes

**Figure 10:**
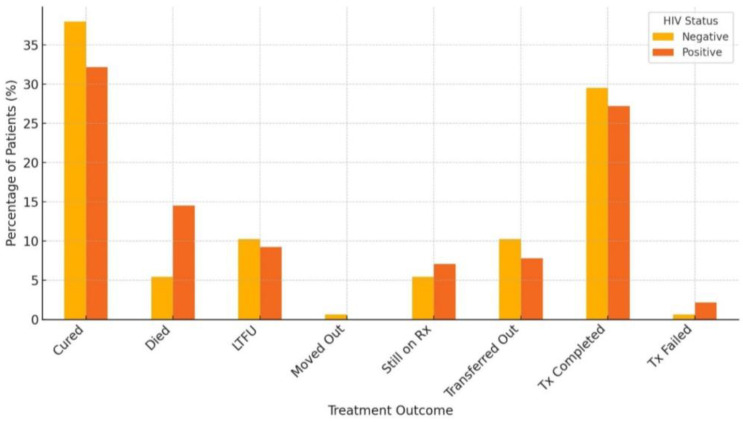
Impact of HIV status on treatment outcomes.

A higher percentage of HIV-negative patients (37.95%) were cured compared to HIV-positive patients (32.16%). The death rate is significantly higher among HIV-positive patients (14.49%) compared to HIV-negative patients (5.42%); with the treatment completed category, the percentage of patients who completed treatment is slightly higher among HIV-negative patients (29.52%) than among HIV-positive patients (27.21%) (Figure 10). This analysis suggests that HIV-positive patients are more likely to experience adverse outcomes, such as death, compared to HIV-negative patients.

### 3.7. Factors Influencing Treatment Outcomes

Using the regression analysis, multiple factors simultaneously influence treatment outcomes, including HIV status, gender, and education. The coefficient for HIV status is 0.41810, indicating that being HIV-positive is associated with a slight increase in the treatment outcome score, which suggests worse outcomes (since higher scores correspond to worse outcomes like “Died” or “Treatment Failed”). The *p*-value for HIV status is 0.0600, which is close to the 0.05 threshold, indicating a marginally significant impact on treatment outcomes. The coefficient for age is negative but very close to zero (−0.0071), suggesting that age has a negligible impact on treatment outcomes in this model. The *p*-value is 0.3420, indicating that age is not a significant predictor of treatment outcomes in this dataset. With gender, the coefficient for being male is 0.36410, suggesting that male patients might have slightly worse outcomes than female patients, but this is not statistically significant (*p* = 0.092). The *p*-values for primary and tertiary education are 0.0340 and 0.0050, respectively, showing significant predictors of treatment outcomes. This analysis shows that HIV status, gender, and education level, particularly tertiary education, influence treatment outcomes, with education being a more significant factor.

### 3.8. Predictors of DR-TB Successful Treatment Outcome Using a Decision Tree Classifier (Supervised Machine Learning Algorithm)

**Figure A1:**
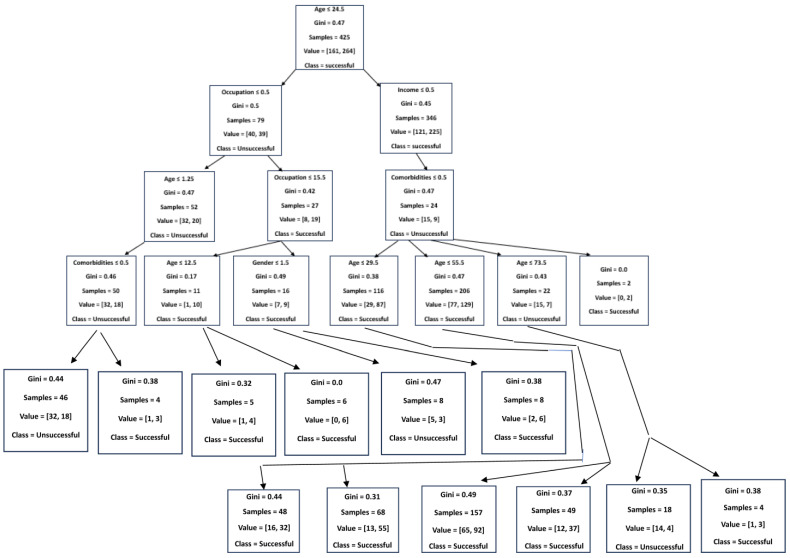
Detailed optimized decision tree for predicting successful treatment outcomes model.

The decision tree model that predicts successful treatment outcomes based on the factors analyzed is shown in Appendix A (Figure A1). The tree shows how factors such as age, income, gender, and comorbidities influence the likelihood of a successful outcome. Each node in the tree represents a decision point based on one of these factors, and the branches show how the data are split based on the values of these factors. The leaf nodes at the bottom represent the final prediction, with the color indicating the likelihood of success or failure.

The model achieved an accuracy of approximately 69%, meaning that it correctly predicted the treatment outcome for about 69% of the cases. The model performs well in predicting successful outcomes but is less effective at predicting unsuccessful ones, as indicated by the lower recall for the unsuccessful class. The model performs well when predicting successful treatment outcomes, as shown by the high recall (92%) and a balanced F1-score (78%). The model performs poorly in predicting unsuccessful outcomes, as indicated by the low recall (31%) and low F1-score (43%). This means that the model misses many actual unsuccessful outcomes. This discrepancy in performance could be due to class imbalance; if the dataset has many more successful cases than unsuccessful ones, the model will naturally perform better on the more frequent class (successful outcomes). It may also suggest that the features used in the model (e.g., age, income, comorbidities) are more predictive of success but less predictive of failure.

Age is the primary factor determining successful treatment, with younger patients (≤24.5) being more scrutinized based on their occupation and comorbidities. Income plays a significant role in older patients (>24.5), where those with income generally have better outcomes. Comorbidities are a consistent factor influencing outcomes across different age and income groups. Gender and Specific Comorbidities further refine the predictions, highlighting more granular relationships between these factors and treatment outcomes.

The top predictors of treatment failure, according to the decision tree, include young age, certain occupations (such as students or prisoners), presence of comorbidities, low income, and age extremes. These factors are strongly associated with a higher likelihood of unsuccessful treatment outcomes, suggesting that patients with these characteristics might require additional support and targeted interventions to improve their chances of success.

In conclusion, the decision tree model performs very well in predicting successful DR-TB treatment outcomes but struggles with unsuccessful outcomes due to low recall and low F1-score. Improvements can be made through techniques like balancing the dataset or adjusting class weights to improve the model’s performance for the unsuccessful class.

## 4. Discussion

Studies have indicated poor outcomes and terrifying high mortality rates among PLHIV coinfected with DR-TB. HIV is also responsible for all forms of MDR and XDR-TB epidemics or outbreaks. TB in PLHIV is mostly smear-negative, and late diagnosis caused by scarcity of rapid diagnostic tests and appropriate treatment has resulted in high mortality in PLHIV. These twin epidemics of DR-TB and HIV have made SSA and Eastern European regions hotbed of fatal human syndemics [18,25,55,56] apart from different levels of exposure in various regions of the world over the last decade, resulting in poor treatment outcomes and high fatality. The difference between the epidemic of HIV infection in the European region and SSA is that while it is concentrated within high-risk groups in the European region, the general population of the SSA has borne the brunt [18].

This study showed a predominance of both RR-TB and MDR-TB in patients who are unemployed and within the economically active age with a 45.0% prevalence, while the proportion of MDR-TB was 42.5%. The 19–35 age group had the highest concentration of RR-TB cases at 41.5%, followed by the age group 36–50 with 32.7% prevalence. The age distribution revealed that the incidence of DR-TB was highest among patients aged 19–35 years, followed by the 36–50 group. This suggests the disease is prevalent in the more economically productive age group. The higher incidence of resistance in younger patients might be attributed to their reluctance to adhere to prescribed medication regimens. A similar pattern was reported by Seloma et al. [57], with most of the DR-TB patients falling within the economically productive age range of 20–39 years, comprising 48.1% of cases. This trend suggests that DR-TB is particularly prevalent among individuals and is vital to the workforce and economic productivity. The prevalence of DR-TB among economically active age groups directly impacts workforce productivity, leading to reduced economic output [5] and increased healthcare costs due to the need for more complex treatment regimens, prolonged hospital stays, and additional healthcare interventions. This financial burden can strain public health systems, particularly in low-resource settings.

The study had 56.1% male participants with DR-TB than 43.9% of female patients. The finding is consistent with the findings from other studies, which report that more men than women are infected with TB or DR-TB [57,58]. This is further reinforced by the WHO 2020 Global Report, which proved that adult men bear the greatest burden of TB, accounting for 56% of all cases, while women and children were 33% and 11% respectively [6].

About three-quarters (76.3%) of the cases of DR-TB in this study were unemployed and had no form of income. Similar findings were found in studies conducted in SSA, including other provinces in South Africa. Several studies in South Africa and SSA have found high rates of unemployment among patients with DR-TB. In a study in the Eastern Cape and Western Cape provinces of South Africa, most patients with MDR-TB were either unemployed or had jobs paying close to minimum wage before their illness [59]. In another retrospective study in Limpopo province, South Africa, the authors attributed their results partially to the high unemployment rate in the predominantly rural province [57]. Such findings underpin the close association between TB and poverty since people with no or low income tend to be vulnerable due to their exposure to different social and health conditions [2]. A review of the socio-economic drivers of DR-TB in Africa identified unemployment as a common risk factor for DR-TB and poor treatment outcomes. Poverty and unemployment create barriers to accessing care and adhering to treatment [60].

The prevalence of DR-TB among unemployed individuals in South Africa is notably high, and studies indicate similar trends in other regions with varying prevalence rates. In Poland, a study observed that over 60% of patients with DR-TB were unemployed. The study emphasized that unemployment is a significant risk factor for poorer treatment outcomes and increased mortality among TB patients [61]. In Thailand, a study reported that unemployed individuals represented a significant percentage of TB cases, indicating that unemployment is a common risk factor for TB across different regions [62].

Several outcomes of DR-TB therapy, including cured, treatment completed, successful outcomes, treatment failure, LTFU, and death, are important to evaluate the effectiveness of such therapy [5,63]. Among the 456 patients with DR-TB reviewed during the study period, 33.77% had been declared cured, 29.17% had completed treatment, 9.43% were LTFU, and 10.96% had died. The findings of our study report a successful treatment outcome rate of 61.4%, which is below the targets set by the National Strategic Plan and the World Health Organization (WHO). This result agrees with the previous studies conducted in another rural town of Eastern Cape with 62% and 65.8% rates [64,65], Limpopo, 57.9% [57], Ghana, 68.46% [66], and North-Central Nigeria, 67.4% [67]. The successful treatment outcome rate is higher than that reported in Abuja, Nigeria, which is 48.8% [68] but lower compared to 88.4% in Northwest Ethiopia [63], 80.7% in North-eastern Ethiopia [69], and 78.6% in Cameroon [70]. The disparity in treatment outcomes among these regions could be attributed to differences in number of study participants, sociodemographic characteristics, cultural practices, and socio-economic status. These results underscore the significance of continued support and interventions for patients undergoing DR-TB treatment, especially for those at risk of losing follow-up or experiencing treatment failure. Addressing socio-economic barriers, improving patient education, and ensuring regular access to health care are crucial for improving treatment outcomes in this vulnerable population.

Although our study had a similar successful treatment rate to that conducted in Limpopo Province, the mortality rate and proportions of patients LTFU were 16.1% and 20.6% [57], respectively. In comparison, our study recorded lower figures of 10.96% and 9.43%, respectively, among the DR-TB participants. Various reasons highlighted to be associated with LTFU of DR-TB patients include untraceable residential address, inability to be located or contacted, death after diagnosis, side effects of medications, lack of knowledge of disease severity, alcoholism, social stigma, difficulty in transportation, long distance to health facility, religious beliefs, negative predisposition toward treatment, poverty and lack of family and social support [71,72,73].

The COVID-19 pandemic had a devastating impact on the treatment outcomes of DR-TB in various regions of Africa, including South Africa. In Gabon, the number of newly detected TB cases fell by 80% in 2020 compared to the previous year. Botswana reported a 20% decline, and Lesotho reported a 35% decrease. Across Africa, 28% fewer patients with DR-TB were detected in 2020 compared to 2019. In South Africa, which has the largest number of DR-TB cases in the continent, 48% fewer people with drug-resistant TB were detected in 2020 compared to 2019. Globally, deaths from TB rose for the first time in a decade. In 2020, Africa reported 549,000 deaths, an increase of around 2000 over 2019. The disruptions of health services by the COVID-19 pandemic also led to a reduction in the number of newly detected TB cases in high-burden African countries [74]. According to the surveillance data analysis by Abdul et al. [75] in Gabon, the COVID-19 pandemic substantially disrupted the case-finding approach dynamics in RR-TB. Consequently, it reduced the number of patients screened between 2020 and 2021.

In our study, DR-TB-HIV coinfected patients have a higher risk of having an unsuccessful treatment outcome compared to non-coinfected patients. The mortality rate is significantly higher among DR-TB/HIV coinfected patients (14.49%) as compared to HIV-negative patients (5.42%). In the treatment completed category, the percentage of patients who completed treatment is slightly higher among HIV-negative patients (29.52%) than among HIV-positive patients (27.21%). This suggests that HIV-positive patients are more likely to experience adverse outcomes, such as death, compared to HIV-negative patients, although regression analysis indicated a marginally significant impact on treatment outcomes. A similar trend was seen in another South African study [76], with an association between HIV infection and TB mortality evident in their findings. TB/HIV coinfected patients had a 2.7 times higher risk of unsuccessful treatment outcomes, including death, compared to HIV-negative TB patients, according to the findings of a study conducted in Southern Ethiopia. The death rate was 5.6% in coinfected patients compared to 2% in HIV-negative patients [77]. The mortality rate recorded in the TB/HIV-positive patients was higher compared to the TB/HIV-negative patients (12% vs. 5.6%) [78]. A study in Kenya reported that although the proportion of TB/HIV coinfections declined from 32% in 2012 to 24% in 2020, the patients still had lower treatment completion compared to HIV-negative TB patients [61]. There is a synergistic relationship between TB and HIV. TB infection aggravates HIV-associated immunodeficiency, while HIV infection modifies the pathogenesis of TB [61]. HIV-associated immunosuppression increases the risk of death during TB treatment. The lower treatment completion rates in the TB/HIV coinfected patients could be attributed to factors such as high pill consumption and drug toxicity, which resulted in poor adherence to the treatment regimen and adverse outcomes [61]. This study reported a significantly higher mortality rate among DR-TB/HIV coinfected patients, as has been reported by other studies [79,80]. A similar pattern is displayed globally, with TB-associated mortality in coinfected patients thrice higher than those without HIV coinfection [17]. Different reasons that account for this could be the delayed diagnosis of HIV, unavailability/inaccessibility of ART medications, sub-optimal concentrations of TB medications, malabsorption of TB medications, drug interactions, and variation in the clinical manifestation of TB [14,21,81]. These findings consistently show that HIV coinfection is associated with higher mortality and lower treatment completion rates among TB patients. Hence, TB/HIV coinfected patients may require longer than standard therapy to avoid relapses or treatment failure.

This study applied a decision tree classifier as a supervised machine learning method to determine predicted treatment outcomes and risk factors associated with DR-TB with HIV coinfections. The model performs well when predicting successful treatment outcomes, as shown by the high recall (92%) and a balanced F1-score (78%) (ranging between 67.5% and 73.4%). The model performs poorly in predicting unsuccessful outcomes, as indicated by the low recall (31%) and low F1-score (43%). This means that the model missed many actual unsuccessful outcomes. The study of Balogun et al. [82] applied five machine learning algorithm, including a decision tree (DT), to predict and determine treatment outcomes and risk factors associated with TB disease. The overall classification showed that all the classification methods performed well in classifying the TB treatment outcome (ranging between 67.5% and 73.4%). Elhag [30] reported that the DT model was more accurate than the artificial neural network (ANN) when used to predict and classify tuberculosis cases in the United States of America using tuberculosis case data. Kalhori et al. [83] explored the use of machine learning to predict the outcome of a course of TB treatment. Using a dataset of 6450 TB incidence from Iran in 2005, a comparison of six classifiers, including DT, Bayesian networks, logistic regression (LR), multi-layer perceptron (MLP), Radial Basis Function, and support vector machine (SVM), was made. The DT model presented the best performance with 97% of Area Under the Curve (AUC) Receiver Operating Characteristics (ROC). Using DT in our study, age was the primary factor determining successful treatment, with younger patients (≤24.5) being more scrutinized based on their occupation and comorbidities, with income playing a significant role in older patients (>24.5). In contrast, those with income generally have better outcomes. Comorbidities are a consistent factor influencing outcomes across different age and income groups. Gender and specific comorbidities further refine the predictions, highlighting more granular relationships between these factors and treatment outcomes. Balogun et al. [82], on the other hand, identified age and length of stay as significant risk factors, while gender was not a significant risk factor for TB patients.

Public policy in South Africa targeting TB and HIV coinfection has led to significant improvements in access to ART for HIV-positive individuals, which has been crucial in reducing TB incidence and mortality. Evidence from the literature indicated that the provision of ART led to substantial reductions in TB incidence by approximately 20% [84]) and improved treatment outcomes for coinfected patients. The provision of isoniazid preventive therapy (IPT), scaled up in the mid-2000s, also reduced the risk of developing TB in PLHIV [85,86,87]. However, ongoing challenges related to historical inequalities, stigma, and resource allocation require sustained attention. Broader public health implications emerge from the findings, particularly in high-burden, resource-limited settings like South Africa, where DR-TB’s prevalence among economically active individuals poses significant challenges to workforce productivity and economic stability. Interventions that address socio-economic determinants of health, such as improving access to healthcare, providing social support, and strengthening patient education, are essential for improving outcomes. Continued investment in integrated healthcare services and community engagement will be crucial for further progress in reducing stigma and encouraging individuals to seek timely care, thereby combating these intertwined epidemics. Gender-sensitive strategies that recognize the unique challenges faced by women in accessing care should be incorporated into health policy, and the analysis of disaggregated data by gender should be carried out to help identify specific trends and barriers affecting different populations, thereby informing targeted interventions. This approach would lead to more equitable health service delivery. Global health policies by the WHO and United Nations should foster collaboration between countries facing similar challenges with TB/HIV coinfection and share best practices and resources to enhance the effectiveness of interventions to reduce the burden of these diseases.

## 5. Conclusions

The burden of TB/HIV coinfection was associated with a higher mortality rate and LTFU. Our findings support the need for systematic HIV testing when initiating TB treatment and targeted interventions toward TB treatment completion and reduction of mortality. Early identification of factors associated with different treatment outcomes is imperative for clinicians, researchers, and policymakers to implement appropriate strategies and measures to treat and manage the TB-infected population in the Eastern Cape. Our findings showed the utility of a decision tree classifier as a model to predict DR-TB patients’ treatment outcomes with a high recall of 92%. Machine learning models can effectively predict patients’ treatment outcomes, highlighting their potential to improve clinical decision-making and patient care. Further investigation is needed using large datasets and more factors to validate this valuable treatment outcome prediction tool.

Our study highlights both successes and gaps in managing DR-TB and HIV coinfection, suggesting that leveraging machine learning, addressing socio-economic barriers, and building resilient healthcare systems can significantly improve outcomes and reduce mortality in this vulnerable population. The study provides a critical understanding of the treatment outcomes of patients with DR-TB and HIV coinfection, particularly in the OR Tambo district, with a treatment success rate of 61.4%, which aligns with global trends but underscores ongoing challenges. The significant reduction in the number of cured patients during the COVID-19 pandemic compared to the pre-pandemic period highlights the detrimental impact of global health crises on managing chronic diseases like TB. This decline, along with the increased number of patients still on treatment, emphasizes the need for resilient healthcare systems capable of maintaining continuity of care during such crises. Lower treatment success rates and higher mortality among DR-TB/HIV coinfected patients demonstrate the urgent need for integrated care approaches, especially for HIV-positive individuals who face higher risks of adverse outcomes. These findings suggest that earlier HIV diagnosis and prompt initiation of ART are crucial, along with efforts to address the socio-economic barriers, such as unemployment and poor healthcare access, which contribute to patient LTFU and poor outcomes.

The pandemic’s impact on DR-TB treatment outcomes also exposes the fragility of healthcare systems in managing concurrent crises, underscoring the need to strengthen healthcare infrastructure to maintain essential services during emergencies. Future research should focus on refining machine learning models by addressing class imbalances, incorporating more sociodemographic data, and exploring socio-economic factors affecting treatment adherence. Strategized interventions, such as conditional cash transfers and community-based support, may reduce LTFU and improve outcomes. Furthermore, integrating TB and HIV care—through earlier diagnosis and prompt ART initiation—could reduce mortality and enhance treatment success, contributing to global TB control goals.

Furthermore, to meet WHO goals for controlling these twin epidemics by 2030, South Africa must enhance testing and treatment for coinfected individuals while addressing socio-economic disparities that hinder effective healthcare delivery.

## Figures and Tables

**Table 1:** Sociodemographic and clinical characteristics of drug-resistant tuberculosis patients from 2018 to 2020 (N = 456) #.

Characteristics	N	%
Gender		
Male	256	56.1
Female	200	43.9
Age groups (years)		
0–18	32	7.2
19–35	185	41.6
36–50	142	31.9
51–65	65	14.6
>66	21	4.7
Occupation		
Unemployed	331	76.3
Employed (govt. and private)	34	7.8
Student	35	8.1
Pensioner	20	4.6
Grant recipient	8	1.8
Minors	6	1.4
Type of TB		
PTB	446	97.8
EPTB	6	1.3
NR	4	0.9
Type of resistance		
Monoresistance	207	45.4
Polyresistance	237	52.0
NR	12	2.6
Type of drug resistance		
RR	205	45.0
MDR	194	42.5
Pre-XDR	23	5.0
XDR	17	3.7
INH-R	6	1.3
NR	11	2.4
Previous drug history		
New	226	49.6
PT1	178	39.0
PT2	43	9.4
UNK	1	0.2
NR	8	1.75
HIV status		
Positive	281	61.6
Negative	165	36.2
NR	10	2.2
BMI status		
Underweight (<18.5 BMI)	58	25.9
Normal weight (18.5–24.9 BMI)	107	48.0
Overweight (25–29.9 BMI)	39	17.5
Obese (≥30 BMI)	19	8.5

PTB—pulmonary TB; EPTB—extrapulmonary TB; RR—Rifampicin resistance; MDR—multidrug-resistant; XDR—extremely drug-resistant; INH-R—isoniazid-resistant; NR—Not reported; PT1—previously treated with first-line drugs; PT2—previously treated with second-line drugs; UNK—unknown. # Some characteristics did not equal 456 because they were not reported.

**Table 2:** Type of DR-TB categorized according to age groups.

DR-TB Type	Age Groups (Years)				
	0–18	19–35	36–50	51–65	>66
RR	15	85	67	24	14
MDR	12	84	62	31	5
Pre-XDR	3	5	7	7	1
XDR	2	11	1	3	0
INH-R	0	0	5	0	1

RR—Rifampicin resistance; MDR—multidrug-resistant; XDR—extremely drug-resistant; INH-R—isoniazid-resistant.

## Data Availability

Data can be requested from the corresponding author.
